# Limited Value of Bladder Wash Cytology During Follow-Up of Patients With Non-muscle Invasive Bladder Cancer

**DOI:** 10.7759/cureus.40283

**Published:** 2023-06-12

**Authors:** Uwe Bieri, Benedikt Kranzbühler, Marian S Wettstein, Christian D Fankhauser, Basil P Kaufmann, Burkhardt Seifert, Peter K Bode, Cédric Poyet, Daniela Lenggenhager, Thomas Hermanns

**Affiliations:** 1 Department of Urology, University Hospital Zürich, Zürich, CHE; 2 Department of Urology, Luzerner Kantonsspital, Luzern, CHE; 3 Epidemiology, Biostatistics and Prevention Institute, University of Zürich, Zürich, CHE; 4 Department of Pathology and Molecular Pathology, University Hospital Zürich, Zürich, CHE

**Keywords:** bladder cancer, non-muscle invasive bladder cancer, bladder wash cytology, specificity, sensitivity, surveillance, cytology, urinary bladder neoplasm

## Abstract

Aims

We aimed to assess the performance of bladder wash cytology (BWC) in daily clinical practice in a pure follow-up cohort of patients previously diagnosed with non-muscle invasive bladder cancer (NMIBC).

Materials and methods

We analyzed 2064 BWCs derived from 314 patients followed for NMIBC (2003-2016). Follow-up investigations were performed using cystoscopy (CS) in combination with BWC. Patients with suspicious CS and/or positive BWC underwent bladder biopsy or transurethral resection. BWC was considered positive if malignant or suspicious cells were reported. Sensitivity (Sn) and specificity (Sp) were calculated for the entire cohort and separately for low-grade (LG) and high-grade (HG) tumors, and carcinoma in situ (CIS) subgroups.

Results

A total of 95 recurrences were detected, of which only three were detected by BWC alone. Overall, Sn and Sp of BWC were 17.9% and 99.5%, respectively. For LG disease, these numbers were 14.0% and 100%, and for HG disease, these were 22.2% and 99.1%, respectively. For patients with CIS at initial diagnosis, Sn and Sp were 11.0% and 71.4%, respectively. For isolated primary CIS, Sn was 50.0%, and Sp was 98.2%.

Conclusion

Routine use of BWC in the follow-up for NMIBC is of limited value even in HG tumors. In the presence of isolated primary CIS, adjunct BWC might be justified.

## Introduction

Urothelial bladder cancer (BC) represents the 9th most common malignancy overall while being the 13th leading cause of cancer mortality [1]. At the time of diagnosis, the majority of all detected BCs are non-muscle invasive (NMI) [2]. However, high recurrence rates of non-muscle invasive bladder cancer (NMIBC) after transurethral resection and the risk of progression into muscle-invasive disease require regular follow-up. For optimal disease management, it is crucial to detect recurrences early during follow-up, particularly in high-grade (HG) diseases. Current guidelines recommend regular follow-up cystoscopies, representing the gold standard investigation in BC surveillance [3]. However, cystoscopies can miss significant lesions (e.g., early small recurrences, flat tumors, or carcinoma in situ (CIS)) [4]. Therefore, urine cytology is often used as an adjunct test for BC surveillance. The reported overall sensitivity of urinary cytology ranges from 20% to 60% in most studies [5-8]. The sensitivity of urinary cytology is known to be higher in HG compared to LG tumors [9] and in larger tumors [10]. Thus, the sensitivity might also be different in primary tumors (often larger) compared to tumors diagnosed during disease surveillance (often smaller). Most studies [6,11,12] show that urine directly collected from the bladder is superior to voided urine when considering different modes of urinary cytology (bladder wash cytology (BWC) vs. voided urine). This could be crucial, especially in the case of recurrence, where tumors are generally smaller.

Most of the studies assessing the role of urinary cytology consist of heterogeneous cohorts (screening and surveillance), different specimen collection techniques (voided urine cytology and BWC), and different definitions of positive cytology (e.g., malignant, suspicious, atypical, and dysplasia) [13]. All these factors impact the interpretation of results and the comparison of individual studies.

Given that the performance of BWC in a pure follow-up cohort has never been investigated, the true impact of BWC in the setting of BC surveillance is still unknown. Although molecular urinary markers have been intensively investigated in recent years, none of them has yet found their way into clinical practice [14]. Therefore, urinary cytology is still widely used in daily clinical practice but is only recommended in high-risk patients in current guidelines [15,16].

The goal of the present investigation was to assess whether BWC improves BC surveillance in a pure follow-up cohort of patients with previously diagnosed NMIBC.

## Materials and methods

This retrospective investigation was performed with a consecutive series of patients followed for NMIBC with cystoscopy and BWC between 2003 and 2016 in our tertiary care academic center. In our institution, all patients were followed using a combination of flexible cystoscopy and BWC, representing a more comprehensive protocol than recommended by the current European Association of Urology (EAU) guidelines [16]. The local ethics committee approved the study protocol for this analysis (Basec-No. 2016-00158).

Clinical information reviewed from electronic patient records included the patient's age, gender, initial tumor stage and grade, presence of concomitant CIS according to the 2004 World Health Organization, and postoperative Bacille Calmette-Guérin (BCG) instillation treatment status. Additionally, overall follow-up time, results of surveillance BWCs, and simultaneously performed cystoscopies were recorded.

Patients with a positive cystoscopy and/or a BWC containing malignant or suspicious cells underwent bladder biopsy or transurethral resection of bladder tumors (TURBT). Those with both negative cystoscopy and BWC were considered recurrence-free and underwent further follow-up. All cytological examinations were performed by specialized cytopathologists.

The samples were processed as cytospins with subsequent Papanicolaou staining. Cytological reports included reporting of four different categories: no evidence of malignant cells, atypical, suspicious, and malignant cells. BWC was considered positive if malignant or suspicious cells were reported.

Statistical analysis was performed per sample considering each a stochastically independent unit (unit of statistical analysis). Possible scenarios were (1) negative cystoscopy and negative BWC, classified as true negative, (2) positive cystoscopy and positive BWC, (3) negative cystoscopy and positive BWC, or (4) positive cystoscopy and negative BWC. All the cases in these categories (2-4) that were scheduled for surgery were classified based on the result of the confirmatory biopsy/TURBT performed within 30 days. We did not exclude cases without histologic follow-up and considered these cases to be negative.

Sensitivity (Sn) and specificity (Sp) were calculated for the entire cohort and separately for LG and HG tumors. As patients with CIS at initial diagnosis have a higher probability to recur with CIS [17], which can be invisible during cystoscopy, BWC might be particularly helpful in these patients. Therefore, we analyzed Sn and Sp for patients with CIS at primary diagnosis (isolated or concomitant) separately.

## Results

Overall, 2064 cystoscopies with BWC were performed in 314 patients followed for NMIBC between 2003 and 2016. Patient characteristics are shown in Table 1. The cohort consisted of 253 (80.6%) male and 61 (19.4%) female patients. The median age at initial tumor diagnosis was 67 years (range: 24-98 years). A total of 206 patients (65.6%) were followed for Ta tumors, 98 patients (31.2%) for T1 tumors, and 10 patients (3.2%) for isolated primary CIS. Additionally, concomitant CIS was detected in 33 patients (10.5%) with Ta/T1 tumors. LG disease was found in 156 patients (49.7%) and HG disease in 158 patients (50.3%). Postoperative BCG treatment was performed in 151 patients (48.1%). Median patient follow-up was 48 months (range: 1-264). The median number of BWCs performed per patient was four (range: 1-27). During follow-up, 95 recurrences were detected. A total of 75 (79%) cases were histologically confirmed, with 32 (43%) LG cases, 42 (56%) HG cases, and one (1%) case where definitive grading was not feasible due to BCG artifacts. CIS was reported in 11 (17%) cases, and stage progression was reported in 16 (21%) cases, with three (4%) cases progressing to muscle-invasive disease.

**Table 1:** Characteristics of all 314 patients Data are presented as median (range) or number (percent). CIS: carcinoma in situ; LG: low grade; HG: high grade.

	n
Age (years)	67 (24-98)
Overall follow-up (months)	48 (1-264)
Gender	
Male	253 (80.6)
Female	61 (19.4)
Primary bladder tumor stage	
Ta	206 (65.6)
T1	98 (31.2)
CIS	10 (3.2)
Primary bladder tumor grade (2004 Classification)	
LG	156 (49.7)
HG	158 (50.3)
Concomitant CIS	
Yes	33 (10.5)
No	281 (89.5)

As shown in Table 2, recurrences were identified by cystoscopy only in 62 cases (65.3%). Three recurrences (3%) were detected by cytology only. Two of these cases presented with isolated CIS. In 14 cases (14.7%), both cystoscopy and cytology were positive. Two cases (2.1%) were incidental findings after negative cystoscopy and cytology. In one case, a simultaneously performed positive loss of heterozygosity/fluorescence in situ hybridization (LOH/FISH) test led to further investigations with photodynamic diagnostics. The other case was an incidental finding after ureteric reimplantation due to distal ureter stenosis. Fourteen patients (13 with initial HG disease) were further investigated due to atypical cells in BWC (surgeon/patient-driven decision). Two of these patients presented initially with isolated primary CIS and four with CIS at recurrence.

**Table 2:** Mode of detection of recurrence during NMIBC surveillance Data are presented as numbers (percent). NMIBC: non-muscle invasive bladder cancer.

	n
Total recurrences	95
Cystoscopy positive/cytology negative	62 (65.3)
Cystoscopy negative/cytology positive	3 (3.2)
Cystoscopy positive/cytology positive	14 (14.7)
Cystoscopy negative/cytology negative	14 (14.7)
Incidental finding	2 (2.1)

The overall Sn and Sp calculated for BWC were 17.9% and 99.5%, respectively. For LG disease, the Sn and Sp were 14.0% and 100%, and for HG disease, the Sn and Sp were 22.2% and 99.1%, respectively (Table 3). For patients with the presence of CIS (isolated or concomitant) at initial diagnosis, Sn and Sp were 11.0% and 71.4%, respectively. For patients with isolated primary CIS, Sn was 50.0% and Sp was 98.2%.

**Table 3:** Sensitivity and specificity of BWC during surveillance Data are presented as percentages. BWC: bladder wash cytology; LG: low grade; HG: high grade; CIS: carcinoma in situ.

	Sensitivity (%)	Specificity (%)
Overall	17.9	99.5
LG	14.0	100
HG	22.2	99.1
CIS (isolated/concomitant)	11.0	71.5
CIS (isolated primary)	50.0	98.2

For the entire cohort, 688 BWCs had to be performed to detect one additional recurrence that was otherwise missed by cystoscopy. For patients with initial HG tumors or patients with isolated primary CIS, these numbers were 581 or 57, respectively.

## Discussion

Our study fills an important gap in the literature on urinary cytology by exclusively analyzing the value of BWC in a pure surveillance cohort followed by cystoscopy in combination with BWC. Our analysis revealed a low overall Sn of BWC (17.9%) when only focusing strictly on the report of malignant and suspicious cells. Only three of the 19 recurrences missed by cystoscopy were detected by BWC. It is notable that two of these recurrences were detected in patients with isolated CIS at recurrence. This finding was enforced by the results of the subgroup analysis of isolated primary CIS patients, which showed a significantly increased Sn of 50% compared to the overall cohort (17.9%) and even the HG subgroup (22.2%).

Given these findings, the routine use of BWC as an adjunct in the surveillance of NMIBC patients is called into question, but it may be justified in patients with isolated primary CIS.

Data on cytology performance in pure surveillance cohorts are scarce. In 2001, Raitanen et al. reported an overall Sn of 19.2% and an Sp of 98.3% for urinary cytology of voided urine in the follow-up of NMIBC patients [18]. An Sn of 12.5% in the LG subgroup is well comparable to our analysis. Since their HG subgroup only consisted of three cases, the calculated Sn of 100% is rather not representative.

The present study is the only one that examined BWC performance in a pure follow-up cohort. The majority of comparable urinary cytology studies investigated mixed populations. In 2005, Tetu et al. reported an Sn and Sp of 29% and 98%, respectively, in 870 urinary samples from a mixed cohort consisting of patients with an initial diagnosis of NMIBC and patients under surveillance. As in our study, reporting of atypical cells was considered negative. For LG disease, the Sn was 18%, and for HG disease, it was 53% [19]. A systematic review from 2005 showed that the Sn of urine cytology is 27% lower during surveillance compared to studies evaluating mixed populations [9]. These findings were also confirmed in our investigation. Patients presenting with a primary tumor often have an increased tumor volume [10]. This difference will most likely impact the diagnostic performance of cystoscopy and cytology during the initial work-up compared to a follow-up situation where patients are often diagnosed with small recurrences.

Our study showed that routine BWC during surveillance of NMIBC patients was of little value to detect recurrences otherwise missed by cystoscopy. According to our data, the only exception to this is the subgroup of patients with isolated primary CIS, where 57 BWC are required to detect one extra recurrence that would otherwise be missed by cystoscopy, compared to 688 for the entire cohort (factor 12). This could be an indication that adjunct BWC might be justified in the follow-up of this specific subgroup, based on the rationale that flat lesions are most prone to be overseen by cystoscopy.

Our investigation furthermore shows that at least 14 of the missed recurrences were negative on cystoscopy and BWC. These cases all presented with atypical cells in BWC. Reporting of atypical cells was until recently strongly investigator dependent. Therefore, it is possible that a different pathologist would have classified atypical cells as suspicious cells or vice versa. With the introduction of the Paris System for Reporting Urinary Cytology, a reference system that defines clear morphologic criteria to classify urinary specimens, a more uniform and reproducible diagnostic is now available [20,21] and will likely increase the performance of cytology particularly concerning the suspicious and atypical category. It is, therefore, highly possible that the 14 cases classified as atypical would have been classified differently under the new classification system.

Our study was limited by the absence of a gold standard reference since most of the negative cystoscopy and BWC results were not confirmed by a histological examination. Therefore, the calculation of negative and positive predictive values was not feasible and Sn and Sp values must be interpreted with caution. This is a known difficulty, which has previously been described [22,23]. Also, the cytology samples were not centrally reviewed after initial reporting. However, this is standard clinical practice and helps to translate our findings into a routine setting. Furthermore, based on the retrospective nature of our study data, analysis is susceptible to selection bias.

## Conclusions

Our study provides valuable data on urinary cytology, focusing on a pure surveillance cohort of NMIBC. Based on our findings, routine use of BWC in the follow-up for NMIBC is of limited value even in HG tumors. In the presence of isolated CIS at initial diagnosis, adjunct BWC due to a much higher Sn might be justified, but this needs to be further investigated.
